# Activation of Peracetic Acid by CoFe_2_O_4_ for Efficient Degradation of Ofloxacin: Reactive Species and Mechanism

**DOI:** 10.3390/molecules28237906

**Published:** 2023-12-02

**Authors:** Rong Li, Xing Lu, Jinxiang Gao, Yifan Chen, Shunlong Pan

**Affiliations:** School of Environmental Science and Engineering, Nanjing Tech University, Nanjing 211816, China; 202161202058@njtech.edu.cn (R.L.); 202161102009@njtech.edu.cn (X.L.); 202261202027@njtech.edu.cn (J.G.); 202261202055@njtech.edu.cn (Y.C.)

**Keywords:** peroxyacetic acid, cobalt ferrite, organic radicals, singlet oxygen, fluoroquinolone antibiotics

## Abstract

Peroxyacetic acid (PAA)-based advanced oxidation processes (AOPs) have attracted much attention in wastewater treatment by reason of high selectivity, long half-life reactive oxygen species (ROS), and wider applicability. In this study, cobalt ferrite (CoFe_2_O_4_) was applied to activate PAA for the removal of ofloxacin (OFX). The degradation of OFX could reach 83.0% via the CoFe_2_O_4_/PAA system under neutral conditions. The low concentration of co-existing anions and organic matter displayed negligible influence on OFX removal. The contributions of hydroxyl radicals (·OH), organic radicals (R-O·), and other reactive species to OFX degradation in CoFe_2_O_4_/PAA were systematically evaluated. Organic radicals (especially CH_3_C(O)OO·) and singlet oxygen (^1^O_2_) were verified to be the main reactive species leading to OFX destruction. The Co(II)/Co(III) redox cycle occurring on the surface of CoFe_2_O_4_ played a significant role in PAA activation. The catalytic performance of CoFe_2_O_4_ remained above 80% after five cycles. Furthermore, the ecotoxicity of OFX was reduced after treatment with the CoFe_2_O_4_/PAA system. This study will facilitate further research and development of the CoFe_2_O_4_/PAA system as a new strategy for wastewater treatment.

## 1. Introduction

Fluoroquinolones (FQs) are synthetic medicines with a broad antibacterial spectrum and potent antibacterial activity used in medicine and aquaculture. Only 30–70% of FQs are digested and absorbed by organisms and excreted directly into the ecosystem as natural or metabolites [1,2,3]. Currently, FQs have been widely detected in wastewater, surface water, groundwater, and even drinking water with trace concentrations ranging from ng/L to μg/L [4]. FQs have attracted widespread public attention as a new environmental pollutant with the environmental risk of induced resistance genes [5]. Ofloxacin (OFX) is a typical representative of FQs and is hard to degrade in the natural environment [6]. Additionally, OFX could form chelates with some metal ions, resulting in more biotoxicity and resistance to degradation [7]. Therefore, it is essential to efficiently remove OFX from the aqueous environment. Advanced oxidation processes (AOPs) are promising methods for antibiotics in water due to the short reaction time and higher efficiency [8,9,10,11]. However, the availability of conventional AOPs is dependent on ideal pH and less interfering substance.

Peroxyacetic acid (PAA) has been widely utilized as a disinfectant, bleaching agent, sterilizer, oxidizing agent, and polymerization catalyst in the food processing, medical, chemical, and paper industries [12,13]. Compared with conventional disinfectants, PAA shows enhanced bactericidal ability, low pH dependence, flexible operation, and less toxicity via by-products [14]. Aside from its disinfectant properties, PAA possesses a high redox potential of 1.96 V, making it capable of degrading organic pollutants [13]. PAA can also be activated by UV irradiation, heat, and transition metal activation, producing reactive oxygen species (ROS) with high oxidation ability as a result, such as hydroxyl radicals (·OH) and organic radicals (CH_3_C(O)O·, CH_3_C(O)OO·), due to the fact that it has an easily activated O-O bond that is also contained in hydrogen peroxide (H_2_O_2_), peroxydisulfate (PDS), and peroxymonosulfate (PMS) [15,16]. The slight dependence on pH, anti-interference, and less disinfection byproducts are the more interesting features of PAA-based AOPs over conventional AOPs [17]. Moreover, transition metals are considered to be the optimal activation method due to their natural abundance, lack of external energy, and highly catalytic performance [18].

Co(II) is generally considered the most effective activator for PAA among the transition metals (e.g., Co(II), Fe(II), Mn(II), and Cu(II)) [16,19,20]. The mechanism of PAA activation by metal ions is shown in Equations (1) and (2) [21]. However, the poor reusability, secondary pollution, and toxicity of metal catalysts are the main hindrances to such homogeneous activation methods [20,22,23,24]. For this, developing heterogeneous cobalt catalysts for PAA activation is essential. Spinel ferrites are widely used as wave-absorbing materials and nanocomposite films due to their excellent stability and fascinating magnetic properties [25,26,27]. Additionally, they are also a good choice to serve as the catalytic material in AOPs. Recently, cobalt ferrite (CoFe_2_O_4_) has been demonstrated as an excellent PAA activator, owing to its strong structural stability, low metal ion leaching, bimetallic components, and magnetic properties [28,29,30,31,32]. As such, the application of CoFe_2_O_4_ in PAA activation might hold promise for the efficient degradation of FQs in wastewater. Even though the CoFe_2_O_4_/PAA system has been studied for removing pollutants from water, there is limited information on what active species are present in the system and how CoFe_2_O_4_ activates PAA to specifically degrade contaminants.

(1) $$M^{n+}+CH_{3}C(O)OOH\to M^{\left(n+1\right)+}+CH_{3}C(O)O\cdot +{\mathrm{OH}}^{-}$$

(2) $$M^{\left(n+1\right)+}+CH_{3}(O)OOH\to M^{n+}+CH_{3}(O)\mathrm{OO}\cdot +\;H^{+}$$

This study aimed to assess the roles of reactive species of CoFe_2_O_4_/PAA in degrading OFX and further elucidate its catalytic mechanism. Firstly, the degradation efficiency of OFX in the CoFe_2_O_4_/PAA system was explored according to the effects of CoFe_2_O_4_ dosages, PAA concentration, initial pH, and water matrix (common anions and HA). Subsequently, the stability and reusability of CoFe_2_O_4_ on PAA activation were evaluated. Furthermore, the dominant reactive species in the CoFe_2_O_4_/PAA system for OFX removal was identified. Finally, the degradation pathway of OFX and the toxicity of OFX before and after its treatment in the CoFe_2_O_4_/PAA system were proposed.

## 2. Results and Discussion

### 2.1. Characterization of CoFe_2_O_4_

**Figure 1:**
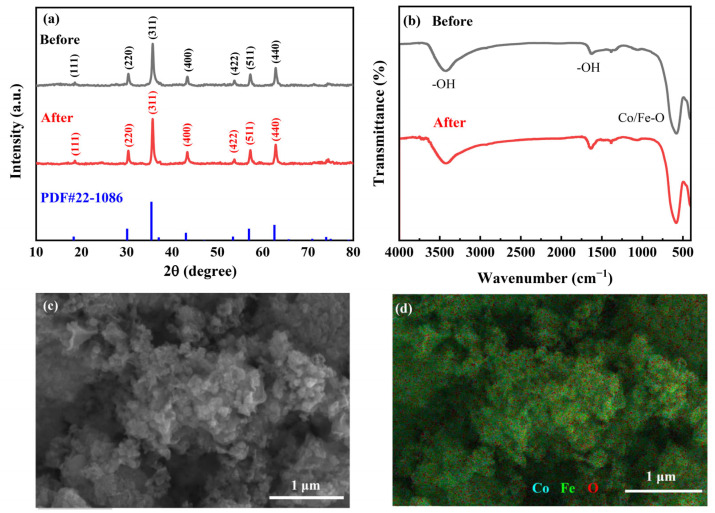
XRD spectra (**a**) and FT-IR spectra (**b**) of CoFe_2_O_4_ before and after the reaction. SEM image of CoFe_2_O_4_ (**c**) with the elemental mapping images of CoFe_2_O_4_ (**d**).

Figure 1a shows the XRD spectra of CoFe_2_O_4_ before and after the reaction. There are seven well-defined peaks in the XRD spectra, which confirm the crystal structure and excellent crystallinity of CoFe_2_O_4_, and the diffraction peaks correspond to the characteristic peaks in the standard spectrum of CoFe_2_O_4_. Moreover, the positions of the characteristic diffraction peaks of CoFe_2_O_4_ did not change before and after the reaction, indicating the great structural stability of CoFe_2_O_4_. The FT-IR spectra of CoFe_2_O_4_ before and after the reaction are presented in Figure 1b. The absorption peaks nearing 3421 cm^−1^ and 1625 cm^−1^ correspond to the stretching and bending vibration of hydroxyl groups, respectively, which are mainly from surface-adsorbed water molecules [33]. Additionally, the peak nearing 580 cm^−1^ can be interpreted as a stretching vibration of metal–oxygen (M-O) and it could further verify the formation of Co/Fe-O [34]. Additionally, it is obvious that CoFe_2_O_4_ exhibits irregular particles with a size of about 100 nm (Figure 1c), and the elemental mapping image (Figure 1d) revealed that Co, Fe, and O were uniformly distributed on the surface of CoFe_2_O_4_.

### 2.2. Performances of OFX Degradation by CoFe_2_O_4_-Activated PAA Oxidation

#### 2.2.1. Degradation of OFX by the System of CoFe_2_O_4_/PAA

**Figure 2:**
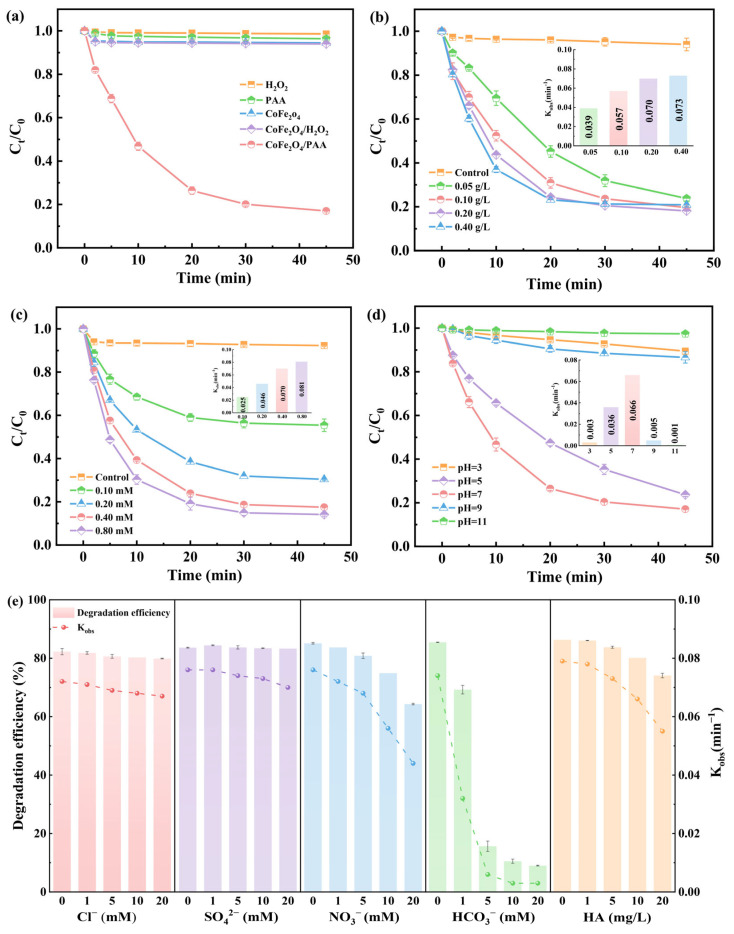
Degradation of OFX in different systems (**a**); the effect of reaction factors on the degradation of OFX in CoFe_2_O_4_/PAA system, the inset figures show the corresponding kinetic constants: CoFe_2_O_4_ dosage (**b**), PAA concentration (**c**), initial pH (**d**), and the effect of water matrix on the degradation of OFX (Cl^−^, SO_4_^2−^, NO_3_^−^, HCO_3_^−^, and HA) (**e**). Experimental conditions: [OFX] = 20 μM, [PAA] = 0.4 mM, [H_2_O_2_] = 0.6 mM, CoFe_2_O_4_ = 0.10 g/L, pH = 7.0, T = 25 °C.

The degradation of OFX under different systems is shown in Figure 2a. OFX was almost not degraded in the system of H_2_O_2_ and PAA alone, indicating that it was negligible to oxidize OFX with H_2_O_2_ and PAA. Only 5.5% and 6.0% of OFX were removed by CoFe_2_O_4_ alone and the CoFe_2_O_4_/H_2_O_2_ system, respectively, suggesting that CoFe_2_O_4_ has weak physical adsorption capability to OFX and trouble activating H_2_O_2_. Compared with other systems, CoFe_2_O_4_ was able to degrade OFX effectively, and the removal rate reached 83.0% after 45 min, which implies that CoFe_2_O_4_ is an effective catalyst for PAA activation. This is due to the activation of PAA adsorption on the surface of CoFe_2_O_4_ and its decomposition to produce highly reactive radicals, which leads to the degradation of organic pollutants [17].

In addition, the decomposition of PAA during the reaction is displayed in Figure S1. Almost all of the PAA decomposed after 45 min. It is noteworthy that the catalytic decomposition of PAA can be accompanied by the production of a large number of fine carbon sources, including methanol (CH_3_OH), acetic acid (CH_3_COOH), and formaldehyde (HCHO) (Equations (3)–(6)) [16,17], and the PAA solution itself contains a certain amount of acetic acid, which can be used in the biological treatment process. Therefore, the CoFe_2_O_4_/PAA process in combination with biological treatment processes has great potential for application in the field of water treatment.

(3) $$CH_{3}C(O)O\cdot \to {\mathrm{CH}}_{3}\cdot +{\mathrm{CO}}_{2}$$

(4) $${\mathrm{CH}}_{3}\cdot +\;O_{2}\to {\mathrm{CH}}_{3}OO\cdot$$

(5) $$2CH_{3}\mathrm{OO}\cdot \to {\mathrm{CH}}_{3}\mathrm{OH}+\mathrm{HCHO}+O_{2}$$

(6) $$CH_{3}\mathrm{OO}\cdot +\;CH_{3}C(O)\mathrm{OO}\cdot \to {\mathrm{CH}}_{3}C(O)\mathrm{OH}+\mathrm{HCHO}+O_{2}$$

#### 2.2.2. Influence of Reaction Factors

The effect of CoFe_2_O_4_ dosage on OFX degradation is shown in Figure 2b. The removal rates of OFX were 76.2%, 80.4%, 81.9%, and 79.1% when the CoFe_2_O_4_ dose was 0.05, 0.10, 0.20, and 0.40 g/L, respectively. At the same time, the corresponding *k*_obs_ increased from 0.039 to 0.073 min^−1^. An increase in surface active sites with increasing CoFe_2_O_4_ accelerates the decomposition of PAA and promotes the generation of ROS. However, CoFe_2_O_4_ itself scavenges ROS when added in excess [35] and causes agglomeration and precipitation, which reduces active sites [21].

The effect of PAA concentration on OFX degradation is shown in Figure 2c. The removal rate of OFX increased from 44.6% to 85.9% when the PAA concentration was raised from 0.1 mM to 0.8 mM, and the *k*_obs_ also grew by 3.2 times. The increase in PAA concentration can fully utilize the active sites of CoFe_2_O_4_ to produce more ROS. Nevertheless, the formation of ROS is relatively slowed when active sites are saturated with PAA because of the high PAA concentration. Furthermore, too much PAA has a quenching impact on ROS [21,36].

The pH is a critical factor affecting OFX removal in the CoFe_2_O_4_/PAA system. The effects of different initial pH on OFX degradation are shown in Figure 2d. In neutral conditions, OFX degraded significantly better than in acidic or alkaline conditions. pH determines the morphology of PAA in aqueous solutions. Since PAA has a *pK*_a_ of 8.2, it mostly resides in its neutral form (PAA^0^) when the pH is below 8.2, and in its deprotonated form (PAA^−^) when the pH is above 8.2 [15]. In addition, pH also affects the surface charge of CoFe_2_O_4_ whose isoelectric point is 6.5 (Figure S2). Therefore, PAA in solution mainly exists in the form of PAA^−^ under alkaline conditions, while the surface of CoFe_2_O_4_ is negatively charged and electrically repulsive, which reduces the chance of contact between PAA and CoFe_2_O_4_. Acidic conditions cause the surface of CoFe_2_O_4_ to become positively charged, and the O-O bond of PAA can easily form a hydrogen bond with H^+^ to become positively charged [37].

#### 2.2.3. Influence of Water Matrix

The widespread presence of anions and dissolved organic matter (DOM) in the aqueous environment could affect OFX degradation by scavenging ROS and forming complexes with metal ions. As shown in Figure 2e, Cl^−^ and SO_4_^2−^ have a slight effect on OFX degradation in the CoFe_2_O_4_/PAA system. As SO_4_^2−^ typically does not react with ROS (·OH, R-O·), its presence in high quantities has no significant impact on OFX removal. According to previous studies, Cl^−^ generally plays two roles in the PAA system: (i) Cl^−^ can react directly with PAA to produce the secondary oxidant HOCl (Equation (7)) [38], and (ii) Cl^−^ also reacts with ROS to form chlorine-containing reactive species (Cl·, HOCl·^−^, Cl_2_^·−^) (Equations (8)–(11)), which have different sensitivities to different pollutants [39].

(7) $${\mathrm{Cl}}^{-}+CH_{3}C(O)OOH\to \mathrm{HOCl}+CH_{3}C(O)O^{-}$$

(8) $${\mathrm{Cl}}^{-}+CH_{3}C(O)\mathrm{OO}\cdot +\;H^{+}\to \mathrm{Cl}\cdot +\;{CH}_{3}C(O)OOH$$

(9) $$\mathrm{Cl}\cdot +H_{2}O↔HOCl\cdot^{-}+\;H^{+}$$

(10) $$HOCl\cdot^{-}↔\mathrm{OH}\cdot +{\mathrm{Cl}}^{-}$$

(11) $$\mathrm{Cl}\cdot +{\mathrm{Cl}}^{-}\to {\mathrm{Cl}}_{2}^{\cdot -}$$

High concentrations of NO_3_^−^ significantly inhibited the degradation of OFX. It is possible that NO_3_^−^ reacts with reactive radicals to generate NO_2_^−^, which also competes with OFX for ROS [40]. Obviously, HCO_3_^−^ strongly suppressed the degradation of OFX. The removal rates of OFX were only 69.3%, 15.7%, 10.5%, and 9.0% when the concentration of HCO_3_^−^ in the system was 1, 5, 10, and 20 mM, respectively. HCO_3_^−^ is a common scavenger of ·OH and hardly reacts with R-O·, therefore the inhibitory effect on OFX degradation is not a competition for reactive radicals [16]. As previously reported, the CoFe_2_O_4_ surface forms a Co-HCO_3_^−^ complex with HCO_3_^−^, which blocks PAA activation [16,41]. Moreover, HCO_3_^−^ is a buffer substance that affects the pH in the reaction system [18], and the weak alkaline conditions are not favorable for OFX degradation by CoFe_2_O_4_/PAA. HA plays a part in inhibiting the removal of OFX in the CoFe_2_O_4_/PAA system, especially at high concentrations of HA. HA is a common radical scavenger [39] and can readily adsorb onto the surface of CoFe_2_O_4_ to form unreactive complexes [30,42].

### 2.3. Reusability and Stability of the CoFe_2_O_4_

**Figure 3:**
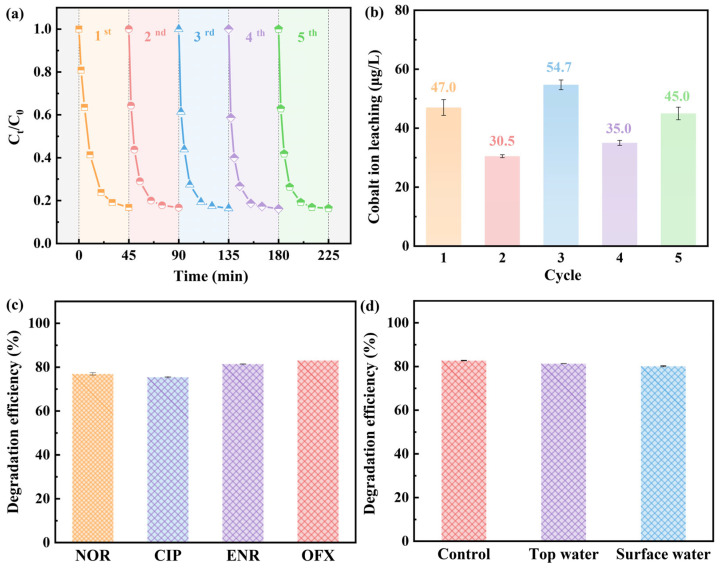
Degradation of OFX (**a**) and the leaching of cobalt ions during cycling experiments (**b**); degradation of different FQs (**c**) and OFX in different water bodies (**d**) by CoFe_2_O_4_/PAA system. Experimental conditions: [OFX] = [NOR] = [CIP] = [ENR] = 20 μM, [PAA] = 0.4 mM, CoFe_2_O_4_ = 0.10 g/L, pH = 7.0, T = 25 °C.

The reusability of CoFe_2_O_4_ was evaluated with cycling experiments, which were carried out under the same reaction system. As can be seen from Figure 3a, the removal efficiency of OFX could still be kept over 80% after five cycles, which indicates that CoFe_2_O_4_ has a stable catalytic performance for PAA. Additionally, the highest leaching amount of cobalt ions was only 0.055 mg/L in cycling experiments (Figure 3b), while iron ions were not detected. Moreover, the XRD and FT-IR analyses of CoFe_2_O_4_ before and after the reaction also further confirmed the structural stability of CoFe_2_O_4_ with regard to PAA activation. The superior catalytic and structural stability of CoFe_2_O_4_ is of great benefit for the application of the CoFe_2_O_4_/PAA system in practice.

### 2.4. Applicability of CoFe_2_O_4_/PAA System

The degradation effect of the CoFe_2_O_4_/PAA system on other three different FQs was investigated and the results are shown in Figure 3c. Norfloxacin (NOR), Ciprofloxacin (CIP), and Enrofloxacin (ENR) were degraded to a great extent after 45 min and the removal rates could reach 77.0%, 75.5%, and 81.5%, respectively, suggesting that the CoFe_2_O_4_/PAA system has the same great degradation performance as other FQs. In addition, the performance of the CoFe_2_O_4_/PAA process for OFX degradation was evaluated in both tap water and surface water. It was pleasantly surprising that the removal efficiency of OFX in tap water and surface water was decreased by only 1.4% and 2.5%, respectively (Figure 3d). The information about these two water types is listed in Table S2. The reason for this slight inhibition may be that coexisting ions and organic matter in actual water can consume ROS. Thereby, the CoFe_2_O_4_/PAA process has promising potential for practical application.

### 2.5. Identification and Analysis of Reactive Species

**Figure 4:**
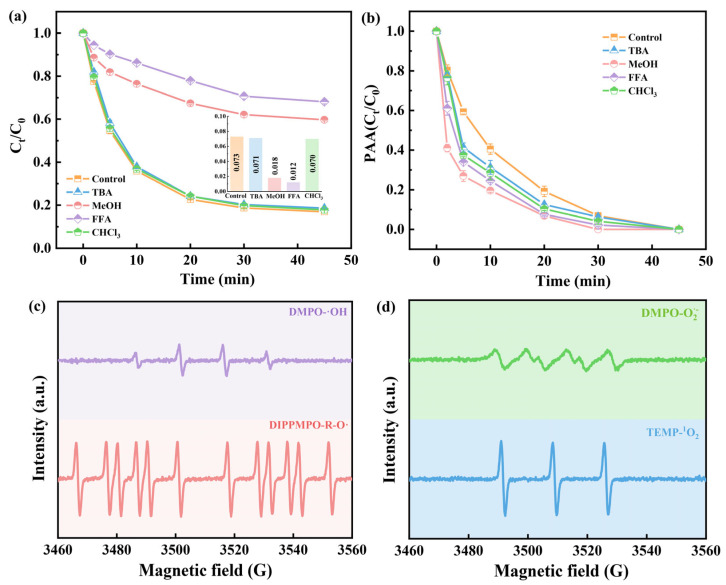
The effect of quenchers on the degradation of OFX (**a**) and the decomposition of PAA (**b**) in CoFe_2_O_4_/PAA system; EPR signal of ROS trapped: ·OH and R-O· (**c**) and O_2_^·−^ and ^1^O_2_ (**d**) in CoFe_2_O_4_/PAA system. Experimental conditions: [OFX] = 20 μM, [PAA] = 0.4 mM, CoFe_2_O_4_ = 0.10 g/L, [TBA] = [MeOH] = 100 mM, [FFA] = [CHCl_3_] = 10 mM, pH = 7.0, T = 25 °C.

It has been reported that various reactive species might be involved in PAA-based AOPs, such as ·OH, O_2_^·−^, ^1^O_2_, R-O· (CH_3_C(O)O·, CH_3_C(O)OO·) and high-valent metal species (Co (IV), Fe (IV)) [16,43]. Therefore, a quenching experiment was conducted to identify ROS produced in the CoFe_2_O_4_/PAA system. Tertiary butyl alcohol (TBA) is a typical ·OH scavenger at a high reaction rate with ·OH (*k*_TBA/·OH_ = 6.0 × 10^8^ M^−1^s^−1^) [44]. As shown in Figure 4a, the addition of excess TBA slightly inhibited the removal of OFX, indicating the minor role of ·OH in the system. MeOH serves as a common scavenger for both ·OH (*k*_MeOH/·OH_ = 9.7 × 10^8^ M^−1^s^−1^) and R-O· in the PAA system [32]. Hence, the contributions of ·OH and R-O· to the degradation of OFX could be distinguished by the use of TBA and MeOH. Obviously, too much MeOH could greatly reduce the removal rate of OFX, which fell from 83.1% to 40.3% (Figure 4a). Thus, the inhibition induced by MeOH was attributed to the scavenging effect of R-O· rather than ·OH. To further verify this, *p*CBA (*k _p_*_CBA/·OH_ = 5.0 × 10^9^ M^−1^s^−1^) and NAP (*k*_NAP/R-O·_= 9.0 × 10^9^ M^−1^s^−1^) were utilized as specific probes for ·OH, R-O· to explore the contribution to OFX degradation [44,45]. As presented in Figure S3, complete removal of NAP occurred within 10 min, whereas only 10% of *p*CBA was degraded after 45 min. This observation evidenced that a large number of R-O· and less ·OH exist in the CoFe_2_O_4_/PAA system.

CHCl_3_ can act as a scavenger of O_2_^·−^, which rapidly reacts with O_2_^·−^ (*k*_CHCl3/O2·−_ = 2.3 × 10^8^ M^−1^s^−1^) [44]. The degradation of OFX was not affected by CHCl_3_, which means O_2_^·−^ had little involvement. Additionally, FFA is widely used as a quencher of ^1^O_2_ (*k*_FFA/1O2_ = 1.2 × 10^8^ M^−1^s^−1^) [16]. According to the results in Figure 4a, the inhibitory effect of FFA on OFX degradation is significantly higher than that of other quenchers, indicating that ^1^O_2_ plays an important role in the degradation process. Meanwhile, the addition of excess quenchers can consume a part of PAA (Figure 4b and Figure S4).

The formation of high-valent metal species like Co(IV) and Fe(IV) in the system could be probed by methyl phenyl sulfoxide (PMSO). PMSO was reported to be converted to methyl phenyl sulfone (PMSO_2_) with high-valent metal species via an oxygen atom transfer route [35,46], in contrast to free radicals, which form hydroxylated products [47]. It turned out that PMSO_2_ occurred in the CoFe_2_O_4_/PAA system, but the conversion rate of PMSO to PMSO_2_ was only 8.0% (Figure S5). Moreover, the removal rate of OFX was only decreased by 2.5% when excess PMSO was introduced (Figure S6). The results demonstrated that high-valent metal species (Co(IV), Fe(IV)) serve a minor role in OFX degradation.

EPR tests were performed to further verify the generation of ROS in the CoFe_2_O_4_/PAA system. DMPO is the spin-trapping agent of ·OH and O_2_^·−^, while DIPPMPO and TEMP serve as the trapping agents for R-O· and ^1^O_2_, respectively [45,48]_._ As shown in Figure 4c,d, the signals of DMPO-·OH (1:2:2:1), DIPPMPO-R-O· (12 lines), DMPO-O_2_^·−^, and TEMP-^1^O_2_ (1:1:1) adducts were detected, demonstrating the existence of ·OH, R-O·, O_2_^·−^, and ^1^O_2_ in the CoFe_2_O_4_/PAA system.

Furthermore, CH_3_C(O)O· and CH_3_C(O)OO· are two critical R-O· species in PAA-based AOPs that are crucial in pollutant degradation. Generally, CH_3_C(O)O· is extremely unstable as a primary radical, susceptible to self-decay to form CH_3_· (*k* = 2.3 × 10^5^ M^−1^s^−1^) (Equation (12)) and is less reactive toward most organic compounds [20,49]. In contrast, CH_3_C(O)OO· is capable of strong oxidation [19]. Thus, CH_3_C(O)OO· is the main R-O· involved in OFX degradation. Based on the above analysis, it can be inferred that R-O· (especially CH_3_C(O)OO·) and ^1^O_2_ are the major reactive species responsible for OFX removal in the CoFe_2_O_4_/PAA system.

(12) $$CH_{3}C(O)O\cdot \;\to \;CH_{3}\cdot +\;CO_{2}$$

### 2.6. Activation Mechanism

**Figure 5:**
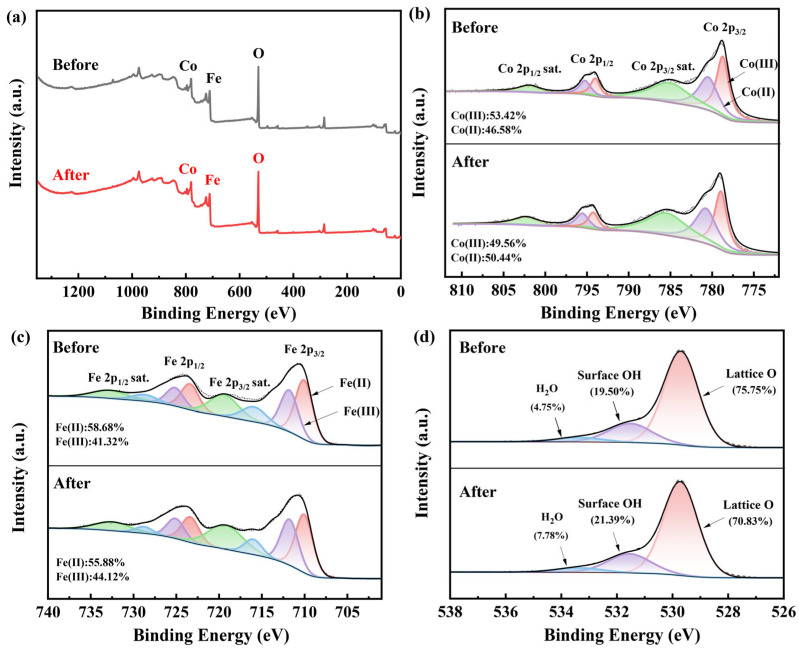
XPS spectra for (**a**) survey scan, (**b**) Co 2p, (**c**) Fe 2p, and (**d**) O 1s of CoFe_2_O_4_ before and after the reaction.

To further illustrate the activation mechanism of CoFe_2_O_4_ on PAA, XPS analysis of CoFe_2_O_4_ before and after the reaction was performed. The XPS full spectrum scans of CoFe_2_O_4_ before and after the reaction are shown in Figure 5a, which reveals the presence of Co, Fe, and O. Figure 5b displays the XPS peak-fitting spectra of Co 2p before and after the reaction of CoFe_2_O_4._ The Co(III) exhibits distinctive peak positions at 778.9 eV (Co 2p_3/2_) and 794.1 eV (Co 2p_1/2_), whereas Co(II) is associated with peak locations at 780.6 eV (Co 2p_3/2_) and 795.4 eV (Co 2p_1/2_). The proportion of Co(III) declined from 53.42% to 49.56%, whereas the Co(II) increased from 46.58% to 50.44%. These observations indicate the presence of a redox cycle involving ≡Co(II)/≡Co(III) on the surface of CoFe_2_O_4_ throughout the reaction [18,45].

Figure 5c shows the XPS peak-fitting spectra of Fe 2p before and after the CoFe_2_O_4_ reaction. The peaks of Fe(II) at Fe 2p_3/2_ and Fe 2p_1/2_ correspond to binding energies of 710.0 eV and 723.4 eV, respectively, while those for Fe(III) are 711.8 eV and 725.1 eV. The proportion of Fe(II) declined from 58.68% to 55.88% and the corresponding increase in Fe(III) from 41.32% to 44.12% suggests the presence of a redox cycle with ≡Fe(III)/≡Fe(II) on the CoFe_2_O_4_. Nevertheless, the involvement of ≡Fe(III)/≡Fe(II) in the activation of PAA is minimal in comparison to ≡Co(II)/≡Co(III) [42]. It should be noticed that the presence of Fe in CoFe_2_O_4_ promotes the transformation of Co(III) to Co(II) (Equation (13)), which improves the electron-transfer capacity of the catalyst [45].

(13) Fe(II)+Co(III)→ Co(II)+Fe(III)

(14) $${\equiv \;Fe}^{3+}+H_{2}O\;\to \;\equiv \;Fe{\mathrm{OH}}^{2+}+H^{+}$$

(15) $$\equiv {\;\mathrm{Co}}^{2+}+H_{2}O\;\to \;{CoOH}^{+}+H^{+}\;\left(\mathrm{slow}\right)$$

(16) $$\equiv {\;\mathrm{Co}}^{2+}+\equiv {\;FeOH}^{2+}\to \;{CoOH}^{+}+{\mathrm{Fe}}^{3+}\;(\mathrm{fast})$$

As shown in Figure 5d, the peaks of lattice oxygen, surface hydroxyl oxygen, and adsorbed oxygen are located at 529.7 eV, 531.5 eV, and 533.2 eV, respectively. The lattice oxygen decreased from 75.75% to 70.83%, while surface hydroxyl oxygen increased from 19.50% to 21.39%, and adsorbed oxygen rose from 4.75% to 7.78%. The decrease in lattice oxygen can be attributed to the reduction of Co(III) to Co(II), while the increase in surface hydroxyl oxygen can be explained by the formation of Co-OH and Fe-OH (Equations (14)–(16)) [30,42].

Therefore, the degradation of OFX in the CoFe_2_O_4_/PAA system was attributed to the generation of reactive species, especially R-O· and ^1^O_2_, and Co played an important role in PAA activation. Based on the above discussion, the activation mechanism of CoFe_2_O_4_ on PAA was proposed. Initially, the surface ≡Co(II) of CoFe_2_O_4_ donates an electron to PAA, which results in the formation of CH_3_C(O)O· and the conversion of ≡Co(II) to ≡Co(III). Subsequently, the generated ≡Co(III) would receive an electron from PAA and recover to ≡Co(II), accompanied by the formation of CH_3_C(O)OO·. Therefore, the ≡Co(II)/≡Co(III) redox cycle is repeated on the catalyst surface to generate R-O· for the degradation of OFX (Equations (17) and (18)). Additionally, the coexisting H_2_O_2_ reacts with R-O· (CH_3_C(O)O·, CH_3_C(O)OO·) to produce HO_2_· (Equations (19) and (20)), which has a weak oxidizing capacity and is prone to forming O_2_^·−^ by deprotonation (Equation (21)). Moreover, ^1^O_2_ could be obtained from the recombination of O_2_^·−^ (Equation (22)). Although the role of Fe in the activation of PAA might be disregarded, the interaction of Fe with Co speeds up the rate of electron transfer at the catalytic interface as well as the adsorption of PAA [42,45].

(17) $$\equiv \;Co(\mathrm{II})+CH_{3}C(O)OOH\;\to \;\equiv \;\mathrm{Co}(\mathrm{III})+CH_{3}C(O)O\cdot +{\;\mathrm{OH}}^{-\;}$$

(18) $$\equiv \;\mathrm{Co}(\mathrm{III})+CH_{3}C(O)OOH\;\to \;\equiv \;\mathrm{Co}(\mathrm{II})+CH_{3}C(O)\mathrm{OO}\cdot +{\;H}^{+}$$

(19) $$H_{2}O_{2}\;+\;{CH}_{3}C(O)O\cdot \to {\mathrm{HO}}_{2}\cdot \;+\;CH_{3}C(O)OH$$

(20) $$H_{2}O_{2}\;+\;{CH}_{3}C(O)\mathrm{OO}\cdot \to {\mathrm{HO}}_{2}\cdot \;+\;CH_{3}C(O)OOH$$

(21) $${\mathrm{HO}}_{2}\cdot \to O_{2}^{\cdot -}+H^{+}$$

(22) 2O2·−+H2O →O21+H2O2+OH−

### 2.7. Degradation Pathway and Toxicity Assessment

**Figure 6:**
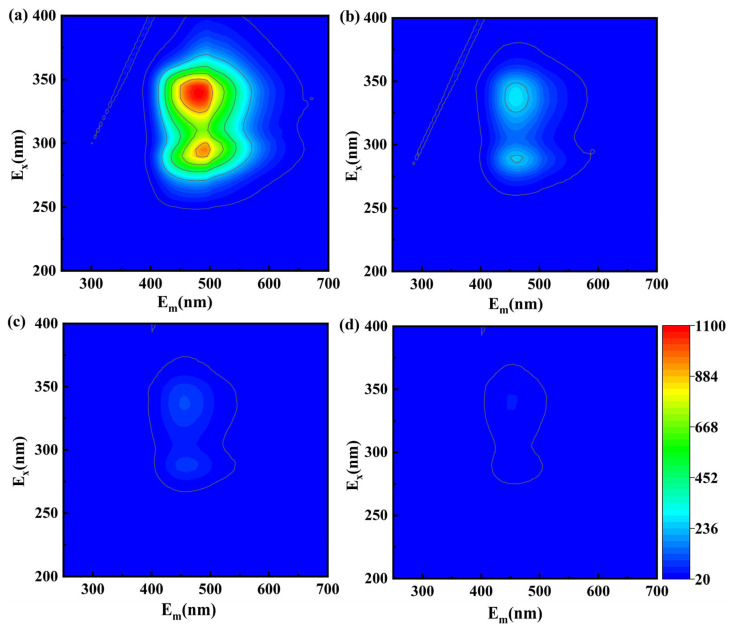
Three-dimensional fluorescence spectra (3D-EEM) at different times during the degradation of OFX by CoFe_2_O_4_/PAA system: (**a**) 0 min, (**b**) 10 min, (**c**) 20 min, (**d**) 45 min. Experimental conditions: [OFX] = 20 μM, [PAA] = 0.4 mM, CoFe_2_O_4_ = 0.10 g/L, pH = 7.0, T = 25 °C.

Three-dimensional fluorescence spectroscopy (3D-EEM) was used to observe the compositional changes of organic matter during OFX degradation and to perform a preliminary analysis of the evolution of the molecular structure. As shown in Figure 6a, OFX showed double fluorescence peaks. The two peaks were located in the ranges of E_X_/E_m_ = (275–300 nm)/(400–575 nm) and E_X_/E_m_ = (300–375 nm)/(400–575 nm), which belong to humic acid-like substances. Meanwhile, it further reflects the aromatic structure of OFX and functional groups such as carboxyl groups (-COOH) and carbonyl groups (-C=O) [50]. As the reaction went on, the intensity of the two fluorescence peaks gradually weakened and the center of the peaks was slightly shifted to the left (Figure 6a–d), implying that the conjugated structure of OFX was continuously destroyed to form intermediate products and that the CoFe_2_O_4_/PAA system could degrade OFX effectively.

**Figure 7:**
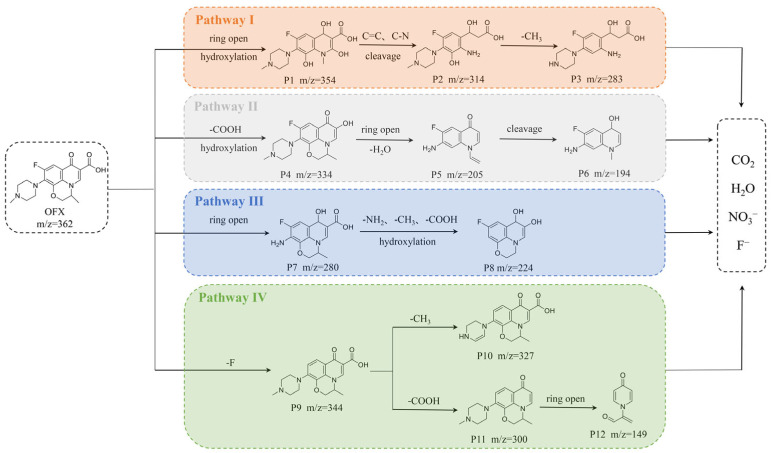
The possible degradation pathway of OFX in CoFe_2_O_4_/PAA system.

The degradation intermediates of OFX in the CoFe_2_O_4_/PAA system were detected by LC-TOF-MS (Figure S7) and summarized in Table S4. Four possible degradation pathways of OFX were proposed based on experimental results and related literature (Figure 7). In pathway I, opening of the oxazine ring and hydroxylation of the quinolone moiety in OFX occurred to form P1 (*m*/*z* = 354), followed by the generation of P2 (*m*/*z* = 314) via the cleavage of C=C and C-N bonds, and was further demethylated to produce P3 (*m*/*z* = 283) [51]. In pathway II, decarboxylation and hydroxylation of the quinolone moiety in OFX formed P4 (*m*/*z* = 334), and P4 was oxidized to produce P5 (*m/z* = 205), which was transformed to P6 (*m*/*z* = 194) via the cleavage of the C=C bond and hydroxylation [52,53]. In pathway III, P7 (*m*/*z* = 280) was obtained by the epoxidation and hydroxylation of piperazine from OFX and was then further converted to P8 (*m*/*z* = 224) via decarboxylation, deamination, and demethylation. As part of pathway IV, OFX was first defluorinated to form P9 (*m*/*z* = 344), followed by the generation of P10 (*m*/*z* = 327) and P11 (*m*/*z* = 300) via demethylation and decarboxylation, respectively, and P12 (*m*/*z* = 149) was obtained by the ring-opening of P11 [51,52]. Eventually, these intermediates are mineralized into inorganic molecules such as CO_2_, H_2_O, NO_3_^−^, and F^−^. As a whole, ring opening, decarboxylation, defluorination, hydroxylation, demethylation, and bond cleavage contribute to OFX degradation.

**Figure 8:**
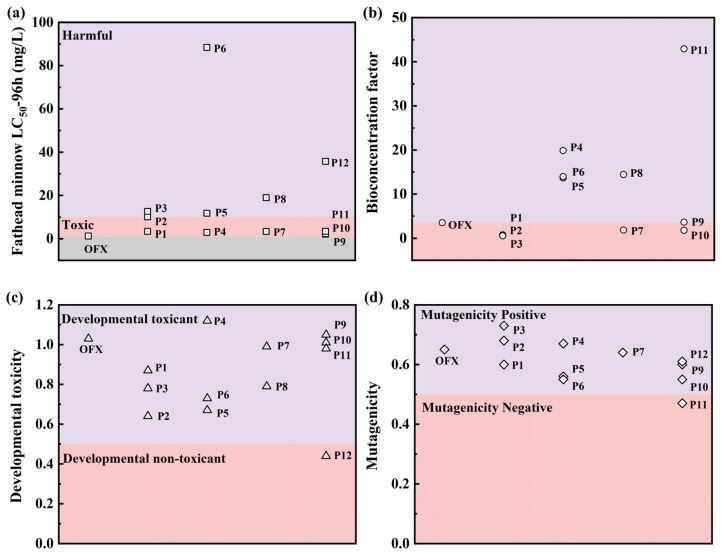
Toxicity assessment of OFX and intermediates: (**a**) Fathead minnow LC_50_ (96 h), (**b**) bioconcentration factor, (**c**) developmental toxicity, and (**d**) Ames mutagenicity.

To evaluate the toxicity variation, toxicity software (T.E.S.T., Version 5.1.2) based on quantitative structure–activity relationship (QSAR) was applied to predict the ecotoxicity of OFX and its detected intermediates, which include acute toxicity, bioconcentration factors, developmental toxicity, and mutagenicity. It is worth mentioning that OFX exhibited toxicity, as seen by its 96 h Fathead minnow LC_50_ value of 1.24 mg/L (Figure 8a). This value was lower than that of the intermediates, suggesting a significant reduction in acute toxicity following degradation. As displayed in Figure 8b, the obtained bioaccumulation factor of OFX and its intermediates indicated that all intermediates except P1, P2, P3, P7, and P10 were lower than OFX. Additionally, the developmental toxicity value of OFX was higher than most of the intermediates (Figure 8c), proving a decrease in developmental toxicity after degradation except for P4 and P9, and P12 even showed developmental non-toxicants. OFX and most of the intermediates were classified as mutagenicity-positive, and P11 was even considered mutagenicity-negative (Figure 8d). After the reaction, it was found that the total toxicity of OFX was lower than before the reaction, indicating that the CoFe_2_O_4_/PAA system offered a high-level potential for toxicity reduction.

## 3. Materials and Methods

### 3.1. Chemicals

Ofloxacin (OFX), Norfloxacin (NOR), Ciprofloxacin (CIP), Enrofloxacin (ENR), Tert-butanol (TBA), Methanol (MeOH), and Trichloromethane (CHCl_3_) were purchased from Macklin Biochemical (Shanghai, China). Furfuryl alcohol (FFA), N, N-diethyl-p-phenylenediamine (DPD), Humic acid (HA), *p*-chlorobenzoic acid (*p*CBA), Naproxen (NAP), Methyl phenyl sulfoxide (PMSO), and Methyl phenyl sulfone (PMSO_2_) were provided by Aladdin Co., Ltd. (Shanghai, China). Acetonitrile (CH_3_CN) and Formic acid (HCOOH) of HPLC grade were purchased from Sinopharm Chemical Reagent Co., Ltd. (Shanghai, China). CoFe_2_O_4_, whose size was around 100 nm, was supplied by Aladdin Co., Ltd. (Shanghai, China). Commercial PAA stock solution was supplied by Kemiou Chemical Reagent (Tianjin, China), and the molar ratio of PAA to H_2_O_2_ in the stock solution was 0.7. All other chemicals and reagents were provided by Sinopharm Chemical Reagent Co., Ltd. The CAS registry number of all chemicals is listed in Table S5. All chemicals were of analytical grade and used without further purification.

### 3.2. Degradation Experiments

Degradation experiments were conducted in a 250 mL glass reactor with shock stirring (Thermostatic shaker SYC-2A, Shanghai Bunting Instrument Co., Shanghai, China) at a speed of 150 rpm. Firstly, 100 mL of 20 μM OFX solution was transferred to the reactor, followed by the addition of a predetermined concentration of PAA, and then the solution’s pH was adjusted with diluted H_2_SO_4_ and NaOH. The experiments were initiated with the addition of CoFe_2_O_4_. All the experiments were maintained at 23 ± 2 °C. Samples were collected within a predetermined time (0, 2, 5, 10, 20, 30, 45 min), and quenched by Na_2_S_2_O_3_, then filtered with a 0.22 μm aqueous filter membrane for analysis.

Different concentrations of anions (Cl^−^, SO_4_^2−^, NO_3_^−^, HCO_3_^−^) and HA were added to the reaction solution to investigate the effect of water matrices on OFX degradation. ROS in the system were identified by radical scavengers (TBA, MeOH, CHCl_3_, FFA) and verified by probe compounds (*p*CBA, NAP, PMSO). NOR, CIP, and ENR were used to evaluate the applicability of CoFe_2_O_4_/PAA systems for FQs. In order to evaluate the stability of the catalytic performance of CoFe_2_O_4_, the used catalyst was desorbed by alkali and then washed with deionized water to neutral and dried at 60 °C. All experiments were conducted at least twice, and the error bar shown in the figure represents the standard deviation between replicates.

The stock solution of OFX (30 mg/L) was obtained by dissolving OFX in pure water with stirring and was stored at 4 °C. The working solution of OFX was diluted from the stock solution. Kinetic analysis of OFX degradation was depicted in Text S1.

### 3.3. Analytical Methods

The surface morphology and chemical composition of CoFe_2_O_4_ were characterized using scanning electron microscopy (SEM, Scios 2 HiVac, FEI, Danville, CA, USA) equipped with energy-dispersive X-ray spectroscopy (EDS). The crystal phase structure of CoFe_2_O_4_ before and after the reaction was determined through an X-ray diffractometer (XRD, SmartLab SE, Rigaku, Japan) with Cu-Ka radiation over the range of 10° to 80°, and Fourier transform infrared spectroscopy (FT-IR, Nicolet iS20, Thermo Scientific, Waltham, MA, USA) was used to determine functional groups. The surface elemental composition of CoFe_2_O_4_ before and after the reaction were analyzed by X-ray photoelectron spectroscopy (XPS, K-Alpha, Thermo Scientific, USA), and the zeta potential of CoFe_2_O_4_ was measured using a Zeta potentiometer (Nano ZS90, Malvern, UK).

The PAA stock solution was calibrated weekly by titration, and the concentrations of peroxide and hydrogen peroxide in the PAA stock solution were determined using iodimetry and potassium permanganate titration, respectively, so as to calculate the concentration of PAA [39]. The residual PAA concentration was determined by N, N-diethyl-p-phenylenediamine (DPD) spectrophotometry [15]. The concentration of OFX, NOR, CIP, ENR, *p*CBA, NAP, PMSO, and PMSO_2_ were detected by high-performance liquid chromatography (HPLC, Agilent 1260, Santa Clara, CA, USA) coupled with an Agilent EC-C18 column and the details of the conditions are presented in Table S6. The concentrations of cobalt and iron ions were analyzed using inductively coupled plasma–atomic emission spectrometry (ICP-AES, ICP-5000, Focused Photonics Inc, Shanghai, China), and pH values were measured by a pH meter (pH-FE28, METTLER TOLEDO, Greifensee, Switzerland). Electron paramagnetic resonance (EPR) tests were carried out to verify the generated ROS using 5,5-dimethyl-1-pyrroline N-oxide (DMPO), 5-Diisopropoxyphosphoryl-5-methyl-1- pyrroline N-oxide (DIPPMPO), and 2,2,6,6-tetramethyl-4-piperidinyl (TEMP) as trapping agents. The detection of three-dimensional fluorescence spectra (3D-EEM) was performed on a fluorescence spectrophotometer (Lengguang F98, Shanghai, China). The intermediates of OFX degradation were determined by LC-QTOF-MS (Agilent 1290-6550, USA) coupled with electrospray ionization (ESI). Meanwhile, the biological toxicity of OFX and its oxidation intermediates was evaluated by the Toxicity Estimation Software Tool (T.E.S.T., Version 5.1.2), which is based on the quantitative structure–activity relationship (QSAR) method.

## 4. Conclusions

In this study, CoFe_2_O_4_ displayed remarkable catalytic performance in the activation of PAA for OFX degradation, and a removal efficiency of 83.0% OFX was achieved within 45 min under neutral conditions. The CoFe_2_O_4/_PAA system exhibited better resistance to anions and HA at low concentrations in water, but NO_3_^−^, HCO_3_^−^, and HA could inhibit OFX degradation at high concentrations. Additionally, CoFe_2_O_4_ showed excellent catalytic performance in cycling experiments and great potential for practical wastewater treatment. R-O· (CH_3_C(O)OO·) and ^1^O_2_ played a dominant role in the degradation of OFX, and the ≡Co(II)/≡Co(III) redox cycle occurring on the surface of CoFe_2_O_4_ during the reaction promoted the decomposition of PAA to generate ROS. Finally, the possible degradation pathways of OFX involved ring opening, bond cleavage, decarboxylation, defluorination, hydroxylation, and demethylation. Toxicity assessment indicated that the CoFe_2_O_4/_PAA system could effectively reduce the biological toxicity of OFX. This study contributes to the practical application of non-homogeneous PAA-based AOPs in wastewater treatment.

## Data Availability

Data are contained within the article and Supplementary Materials.

## Supplementary Materials

The following supporting information can be downloaded at: https://www.mdpi.com/article/10.3390/molecules28237906/s1. Refs. [54,55,56,57,58,59] are cited in Supplementary Materials.
