# Supplementary material for: Activation of Peracetic Acid by CoFe2O4 for Efficient Degradation of Ofloxacin: Reactive Species and Mechanism
Source: Molecules. 2023 Dec 2;28(23):7906. doi: 10.3390/molecules28237906 (PMC10708156; doi:10.3390/molecules28237906)
Supplement: Supplementary file 1 [file molecules-28-07906-s001.zip › molecules-2727200-supplementary.pdf]

Supplementary Material

Activation of Peracetic Acid by $CoFe_2O_4$ for Efficient Degradation of Ofloxacin: Reactive Species and Mechanism

Rong Li, Xing Lu, Jinxiang Gao, Yifan Chen, Shunlong Pan *

School of Environmental Science and Engineering, Nanjing Tech University, Nanjing 211816, China; 202161202058@njtech.edu.cn (R.L.); 202161102009@njtech.edu.cn (X.L.); 202261202027@njtech.edu.cn (J.G.); 202261202055@njtech.edu.cn (Y.C.)
* Correspondence: shunlongpan@njtech.edu.cn

Test S1. Pseudo-first-order kinetic equation.

Table S1. An overview of research on the removal of OFX by ferrites.

Table S2. Quality of tap water and surface water.

Table S3. Change of valence state of elements in $CoFe_2O_4$ before and after reaction by XPS analysis.

Table S4. Intermediates of OFX degradation in $CoFe_2O_4$/PAA system.

Table S5. CAS Registry Number of chemicals.

Table S6. Details of the detection conditions in HPLC for organic compounds.

Table S6. CAS Registry Number of chemicals.

Figure S1. Decomposition of PAA during the reaction.

Figure S2. The zeta potential of $CoFe_2O_4$.

Figure S3. Degradation of NAP and *p*CBA in $CoFe_2O_4$/PAA system.

Figure S4. Effects of quenchers on the decomposition of PAA.

Figure S5. Degradation of PMSO and production of $PMSO_2$ in $CoFe_2O_4$/PAA system.

Figure S6. Effect of PMSO on the degradation of OFX.

Figure S7. LC-TOF-MS chromatograms of OFX and the intermediates in $CoFe_2O_4$/PAA system.

Test S1. Pseudo-first-order kinetic equation

The apparent rate constant (k_{obs}) was calculated by fitting the degradation kinetics of OFX with a pseudo-first-order model:

$$-\ln\left(\frac{C_t}{C_0}\right) = k_{obs}\, t \qquad \text{(S1)}$$

Where, C_0 and C_t represent the concentration of OFX at the initial and a certain reaction time respectively, and t represents the reaction time.

Table S1. An overview of research on the removal of OFX by ferrites.

Catalyst	OFX	Operation conditions	Removal Efficiency	Ref
$Ag_3PO_4/CoFe_2O_4$ (0.5 g/L)	20 mg/L	under visible light; 150 min	95.9%	[1]
$MSm_xFe_{2-x}O_4$ (M = Ni, Co; x = 0, 0.02, 0.06, 0.1) (0.5 g/L)	15 mg/L	$[H_2O_2]$ = 9.8 mM; pH = 2.5; 60 min; under visible light	57%-86%	[2]
$CuFe_2O_4$-Mt (0.4 g/L)	40 mg/L	[PMS] = 2.0 mM; pH = 6.8; 60 min	85.2%	[3]
$CoFe_2O_4$@Bi_2O_3/NiO (0.3 g/L)	10 mg/L	under visible light; 90 min	95.2%	[4]
Cellulose/PANI/$NiFe_2O_4$ (0.5 g/L)	0.05 mM	$[H_2O_2]$ = 8.8 uM; pH = 2.5; 30 min under visible light	87.44%	[5]
$CoFe_2O_4$/WS (1.0 g/L)	20 mg/L	[PMS] = 1.0 g/L; 30 min	92.8%	[6]
$MnFe_2O_4$@C-NH_2 (1.0 g/L)	30 mg/L	$[H_2O_2]$ = 29.4 mM; pH = 3; 180 min	97.4%	[7]

Table S2. Quality of tap water and surface water.

**Category**	**TOC** **（mg/L）**	**COD** **（mg/L）**	**TN** **（mg/L）**
Tap water	0.79	2.31	1.56
Surface water	12.59	39.03	2.31

Table S3. Change of valence state of elements in $CoFe_2O_4$ before and after reaction by XPS analysis.

Elements		Before	After
Co	Co(II)	46.58%	50.44%
	Co(III)	53.42%	49.56%
Fe	Fe(II)	58.68 %	55.88%
	Fe(III)	41.32%	44.12%
O	Lattice O	75.75%	70.83%
	Surface OH	19.50%	21.39%
	H_2O	4.75%	7.78%

Table S4. Intermediates of OFX degradation in $CoFe_2O_4$/PAA system.

Product ID	Molecular weight [M + H⁺]	Molecular formula	Proposed structure
OFX	362	$C_{18}H_{20}O_4N_3F$	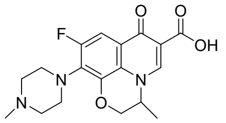
P1	354	$C_{16}H_{20}O_5N_3F$	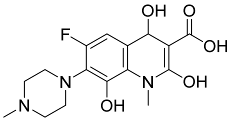
P2	314	$C_{14}H_{20}O_4N_3F$	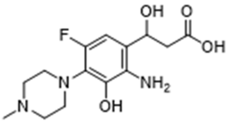
P3	283	$C_{13}H_{18}O_3N_3F$	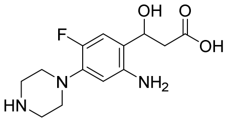
P4	334	$C_{17}H_{20}O_3N_3F$	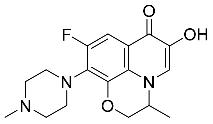
P5	205	$C_{11}H_9ON_2F$	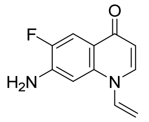
P6	194	$C_{10}H_{11}ON_2F$	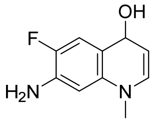
P7	280	$C_{13}H_{13}O_4N_2F$	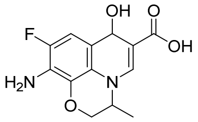
P8	224	$C_{11}H_{10}O_3NF$	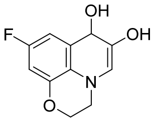
P9	344	$C_{18}H_{21}O_4N_3$	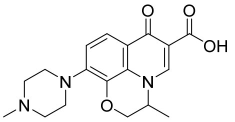

P10	327	$C_{17}H_{17}O_4N_3$	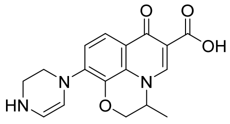
P11	300	$C_{17}H_{21}O_2N_3$	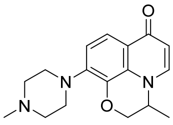
P12	149	$C_8H_7O_2N$	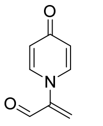

Table S5. CAS Registry Number of chemicals.

Chemicals	CAS	Chemicals	CAS
OFX	82419-36-1	DPD	93-05-0
NOR	70458-96-7	HA	1415-93-6
CIP	85721-33-1	*p*CBA	74-11-3
ENR	93106-60-6	NAP	22204-53-1
TBA	75-65-0	PMSO	1193-82-4
MeOH	67-56-1	$PMSO_2$	3112-85-4
$CHCl_3$	67-66-3	CH_3CN	75-05-8
FFA	98-00-0	HCOOH	64-18-6

Table S6. Details of the detection conditions in HPLC for organic compounds.

Chemicals	Flow rate (mL/min)	Mobile phase	Wavelength (nm)
Ofloxacin (OFX)	1.0	Acetonitrile: 0.1% formic acid (15:85)	288
Norfloxacin (NOR)	1.0	Acetonitrile: 0.1% formic acid (30:70)	275
Ciprofloxacin (CIP)	1.0	Acetonitrile: 0.1% formic acid (30:70)	275
Enrofloxacin (ENR)	1.0	Acetonitrile: 0.1% formic acid (20:80)	278
p-chlorobenzoic acid (*p*CBA)	1.0	Acetonitrile: 0.1% formic acid (40:60)	236
Naproxen (NAP)	1.0	Acetonitrile: 0.1% formic acid (60:40)	231
Methyl phenyl sulfoxide (PMSO)	1.0	Acetonitrile: 0.1% formic acid (25:75)	230
Methyl phenyl sulfone ($PMSO_2$)	1.0	Acetonitrile: 0.1% formic acid (25:75)	215

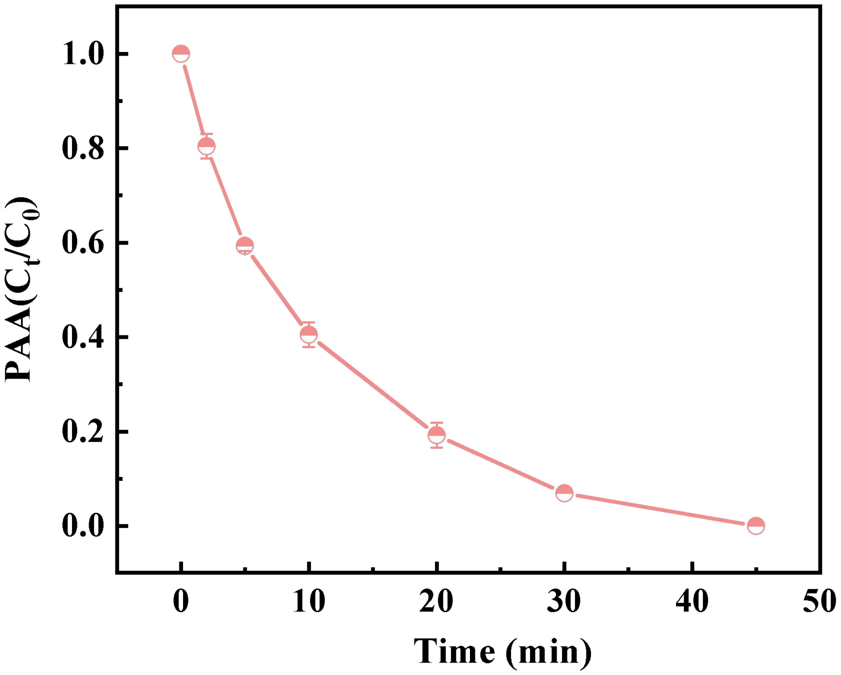

Figure S1. Decomposition of PAA during the reaction. Experimental conditions: [OFX] = 20 μM, $[PAA]_0$ = 0.4 mM, $CoFe_2O_4$ = 0.10 g/L, pH = 7.0, T = 25 °C.

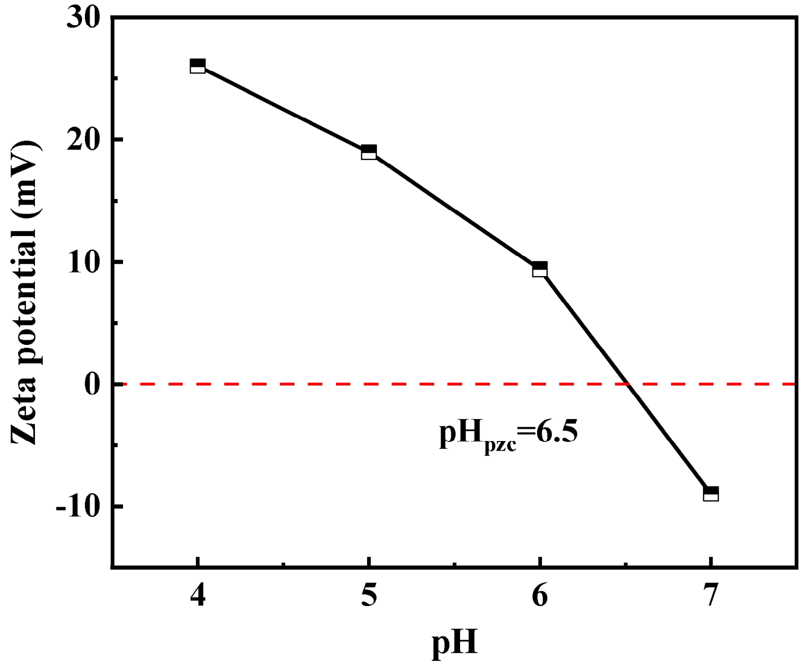

Figure S2. The zeta potential of $CoFe_2O_4$.

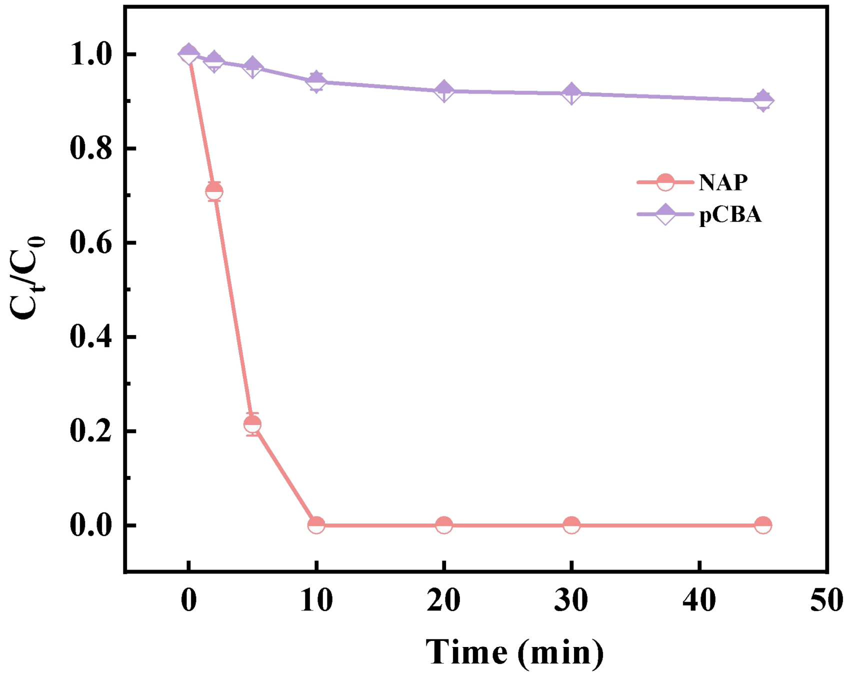

Figure S3. Degradation of NAP and *p*CBA in $CoFe_2O_4$/PAA system. Experimental conditions: [NAP] = [*p*CBA] = 20 μM, $[PAA]_0$ = 0.4 mM, $CoFe_2O_4$ = 0.10 g/L, pH = 7.0, T = 25 ℃.

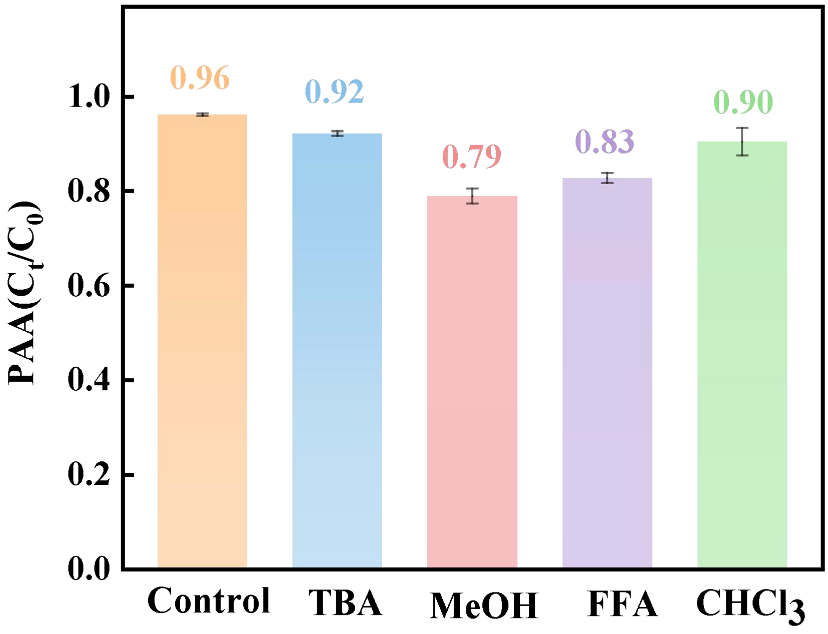

Figure S4. Effects of quenchers on the decomposition of PAA. Experimental conditions: $[PAA]_0 = 0.4$ mM, [TBA] = [MeOH] = 100 mM, [FFA] = $[CHCl_3]$ = 10 mM, pH = 7.0, T = 25 °C.

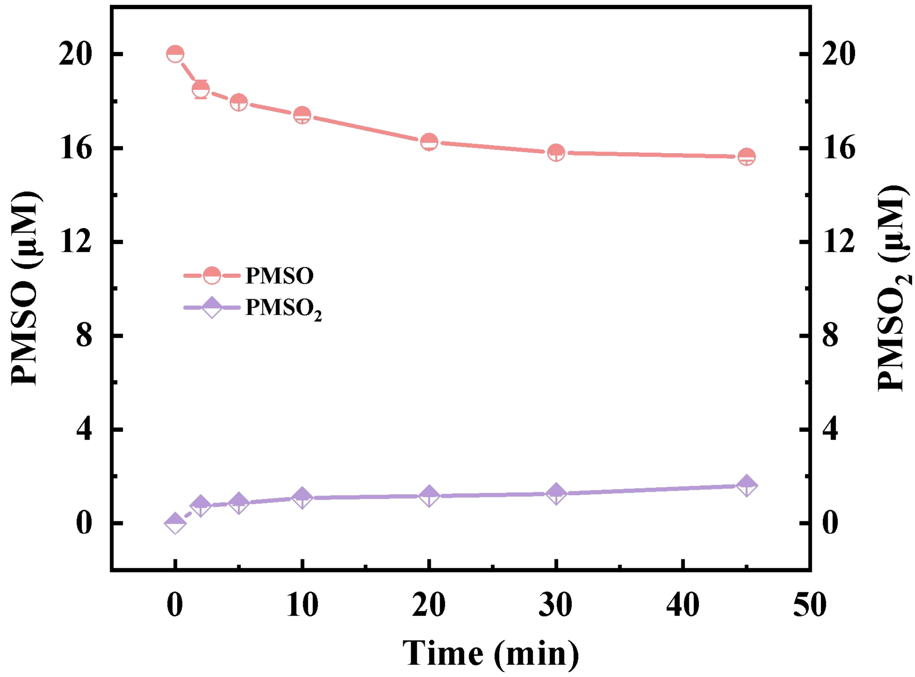

Figure S5. Degradation of PMSO and production of $PMSO_2$ in $CoFe_2O_4$/PAA system. Experimental conditions: [PMSO] = 20 μM, $[PAA]_0$ = 0.4 mM, $CoFe_2O_4$ = 0.10 g/L, pH = 7.0, T = 25 °C.

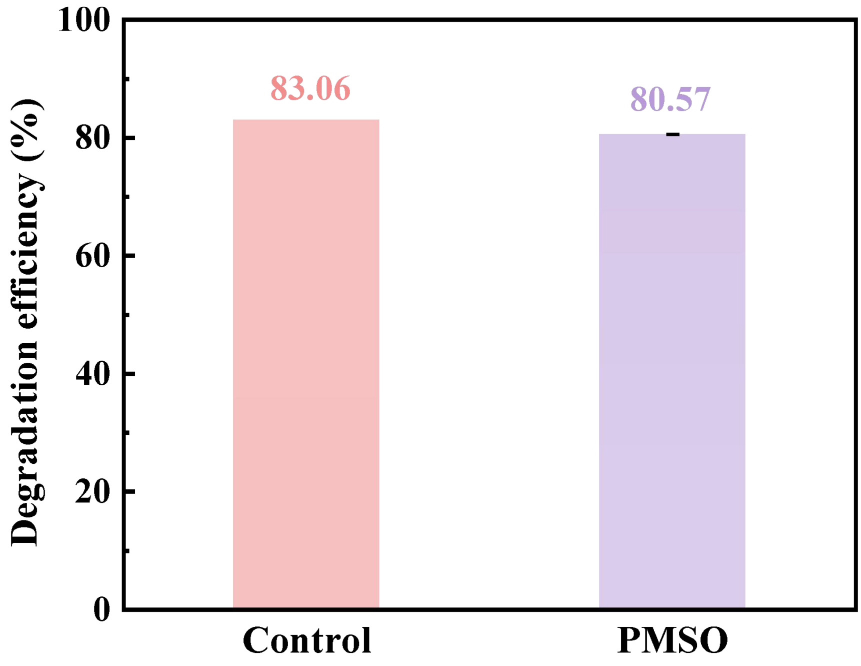

Figure S6. Effect of PMSO on the degradation of OFX. Experimental conditions: [OFX] = 20 μM, [PMSO] = 100 μM, $[PAA]_0$ = 0.4 mM, $CoFe_2O_4$ = 0.10 g/L, pH = 7.0, T = 25 ℃.

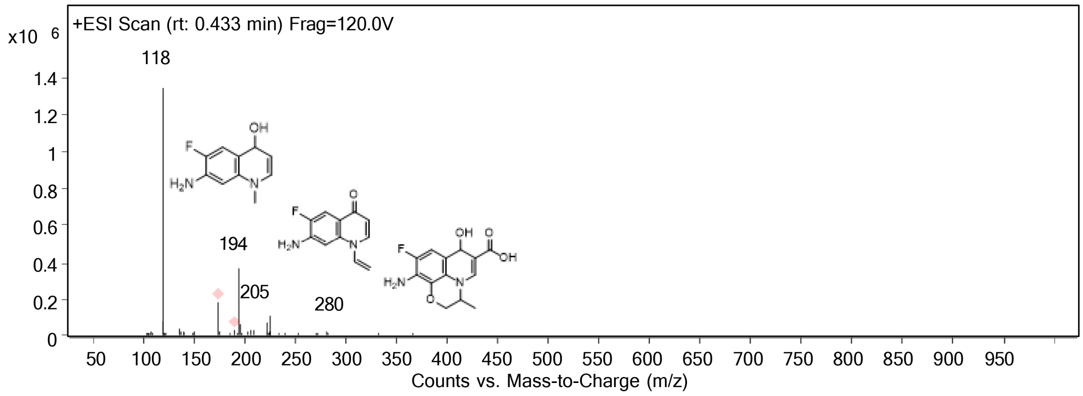
x10 6
+ESI Scan (rt: 0.433 min) Frag=120.0V
118
194
205
280
Counts vs. Mass-to-Charge (m/z)

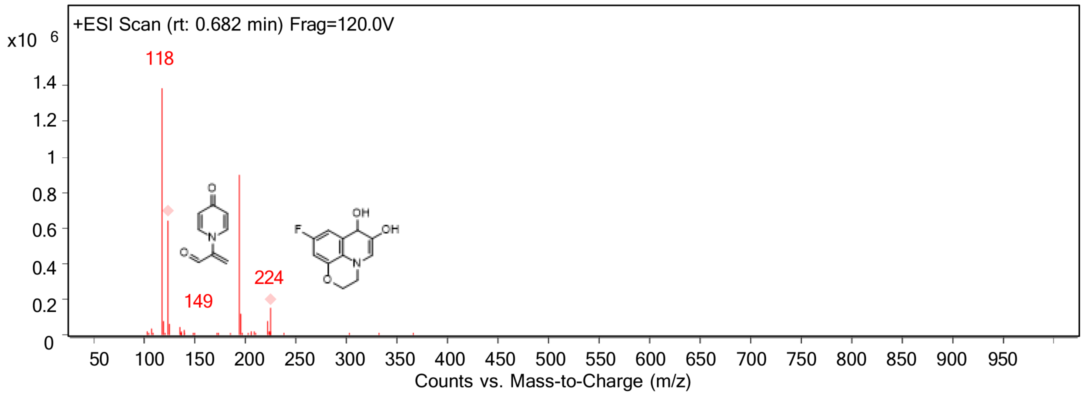
x10 6
+ESI Scan (rt: 0.682 min) Frag=120.0V
118
149
224
Counts vs. Mass-to-Charge (m/z)

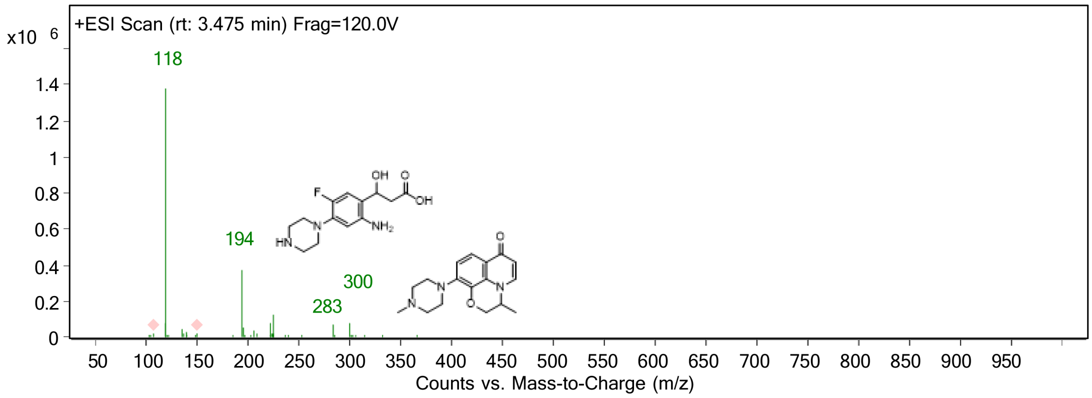
x10 6
+ESI Scan (rt: 3.475 min) Frag=120.0V
118
194
283
300
Counts vs. Mass-to-Charge (m/z)

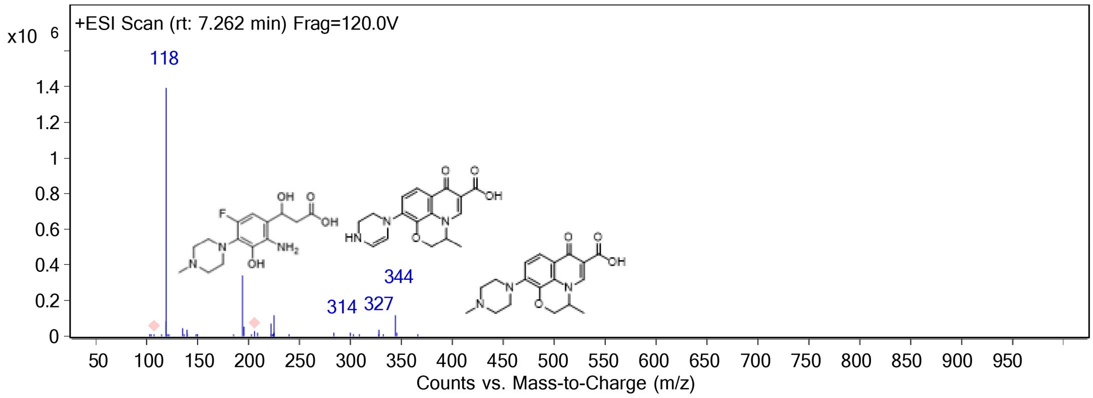
x10 6
+ESI Scan (rt: 7.262 min) Frag=120.0V
118
314
327
344
Counts vs. Mass-to-Charge (m/z)

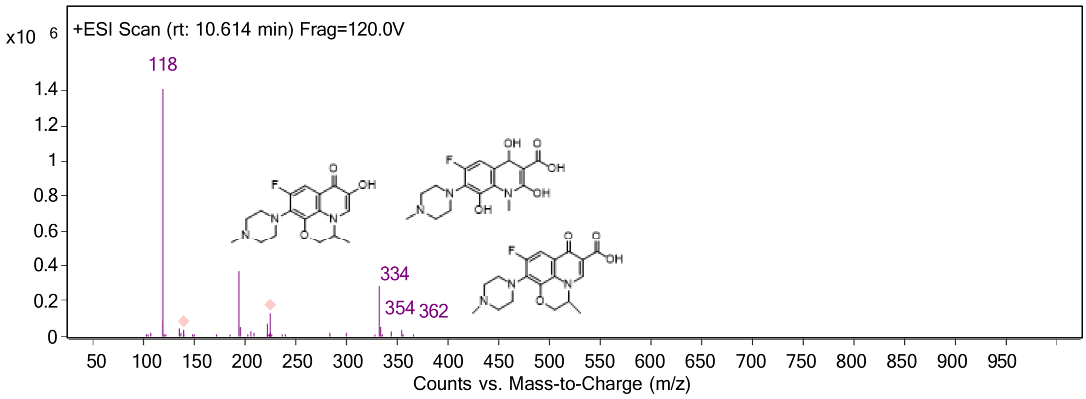

Figure S7. LC-TOF-MS chromatograms of OFX and the intermediates in $CoFe_2O_4$/PAA system.

References

1. Ye, H.; Xia, L.; Wang, Y.; Zhou, H.; Xie, X.; Zuo, G.; Wu, K. Magnetic $Ag_3PO_4/CoFe_2O_4$ Z-scheme heterojunction material for photocatalytic decomposition of ofloxacin. *J. Mater. Sci. Mater. Electron.* **2023**, *34*, 2090.

2. Singh, S.; Kaur, P.; Kumar, V.; Tikoo, K.B.; Singhal, S. Traversing the advantageous role of samarium doped spinel nanoferrites for photocatalytic removal of organic pollutants. *J. Rare Earths.* **2021,** *39*, 781-789.

3. Cao, X.Q.; Xiao, F.; Lyu, Z.W.; Xie, X.Y.; Zhang, Z.X.; Dong, X.; Wang, J.X.; Lyu, X.J.; Zhang, Y.Z.; Liang, Y. $CuFe_2O_4$ supported on montmorillonite to activate peroxymonosulfate for efficient ofloxacin degradation. *J. Water Process. Eng.* **2021**, *44*, 102359.

4. Dhiman, P.; Sharma, G.; Alodhayb, A.N.; Kumar, A.; Rana, G.; Sithole, T.; Alothman, Z.A. Constructing a visible-active $CoFe_2O_4$@Bi_2O_3/NiO nanoheterojunction as magnetically recoverable photocatalyst with boosted ofloxacin degradation efficiency. *Molecules.* **2022**, *27*, 8234.

5. Nitansh; Kaur, P.; Garg, T.; Renu; Deepeka; Kumar, V.; Tikoo, K.; Kaushik, A.; Singhal, S. A fluorescent biomass derived cellulose/PANI/$NiFe_2O_4$ composite for the mitigation of toxic pollutants and Cr(VI) sensing from aqueous environment. *J. Ind. Eng. Chem.* **2023**.

6. Zheng, C.; Wu, J.Y.; Zhang, Y.J.; Yang, Q.; Yang, Y.C. Walnut shell loaded with cobalt ferrite as efficient peroxymonosulfate activator to degrade ofloxacin. *Water Air Soil Pollut.* **2023**, *234*, 305.

7. Qin, H.; Cheng, H.; Li, H.; Wang, Y. Degradation of ofloxacin, amoxicillin and tetracycline antibiotics using magnetic core–shell $MnFe_2O_4$@C-NH_2 as a heterogeneous fenton catalyst. *Chem. Eng. J.* **2020**, *396*, 125304.
